# Potential impact of artificial intelligence on the emerging world order

**DOI:** 10.12688/f1000research.124906.2

**Published:** 2023-10-30

**Authors:** Anupama Vijayakumar

**Affiliations:** 1 Department of Geopolitics and International Relations, Manipal Academy of Higher Education, Manipal, Karnataka, 576104, India

**Keywords:** AI, Technological Diffusion, World Order, Great Power Competition, Fourth Industrial Revolution, Emerging Technologies, Technological Innovation, Great Power Status

## Abstract

The fast-paced diffusion of technologies broadly falling under the umbrella of artificial intelligence (AI) is said to be shaping the emerging world order in international relations (IR). It is expected that the global AI race will pave the way for another rise and fall of great powers in the international system, similar to the impact caused by the three industrial revolutions of the past. The literature in IR identifies three major powers – namely, the United States of America (USA), China, and Russia, as the leading contenders in this AI race. The ongoing AI-enabled fourth industrial revolution is all the more unique due to the markedly different approaches these three powers have adopted for integrating AI into their military, political, and socio-economic spheres. The AI strategies of these countries further reflect their capabilities and intentions towards how they plan on employing the technology to elevate their prestige and power status in the international system. This paper draws from a historiography of the First, Second, and Third Industrial Revolutions to study how technological innovations have altered relative power capabilities of nations, triggering a re-ordering of power hierarchies at a systemic level. Drawing from this understanding, it analyses the nature of AI as an emerging technology and assesses whether it can cause systemic alterations. It critically examines and compares the AI strategies of the USA, China, and Russia as leading contenders in the global AI race and outlines their strengths and weaknesses. It further draws from the Adoption Capacity Theory to argue that the AI race may well be determined by the relative capacity of the major institutions in each of these countries to manage and adapt to the disruptions this technology is bound to bring to the fore.

## Introduction

The world is currently witnessing the Fourth Industrial Revolution ( Schwaab, 2015; Rotatori, Lee, & Sleeva, 2021; Amaresh, 2022). The advent of technologies including artificial intelligence (AI), robotics, big data analytics, and the Internet of Things (IoT) is ushering in a wave of changes across military, political, economic, and societal spectra and forcing a re-think into how activities from individual to global levels have so far been conducted ( Rotatori, Lee, & Sleeva, 2021). These technologies offer tremendous potential to solve problems plaguing the status quo. However, the nature of disruption that they could bring comes with novel challenges whose nature is still unfolding at this stage. These debates have arguably been highlighted the most around AI which has been touted to be the ultimate gamechanger amongst the Fourth Industrial Revolution technologies ( Ayyar, 2016; Del Castillo, 2018; Girasa, 2020).

AI has already crept into activities at various levels to heavily influence human behaviour in tangible or intangible ways. For instance, user behaviour on the internet within platforms ranging from social media to online shopping is heavily conditioned by manipulation through AI algorithms ( Petropoulos, 2022; Rathenau Instituut, 2022). Meanwhile, the technology also learns or evolves through the various patterns it picks up from a person’s online preferences. Law enforcement is another sector where use of AI, particularly through predictive policing is revolutionising traditional methods of preventing and apprehending criminal or terrorist threats ( Verma, 2022; Hunt, 2022). Ethical concerns including those pertaining to the violation of individual privacy and systemic bias have been raised against the practices accompanying AI integration in several instances ( Jenkins & Purves, 2020; Heaven, 2020).

The transformative effects of AI are particularly relevant to understand in the context of geopolitics and international relations (IR). Through its integration into various sectors of the society, the technology is slowly shaping major geopolitical trends ranging from the United States of America (USA)-China technological rivalry to triggering an interest in intelligent warfare among major powers around the world ( Kapetas, 2020). Moreover, these powers seem to view AI as a means to improve their relative position in the international system in economic and military terms ( Alexandre & Miailhe, 2017). The interest in AI, particularly from an international relations point of view, can be seen to be springing primarily from three factors:

♦ The nature and purpose of the technology.
♦ Rapid advances in the field of AI over the past decade.
♦ Historical experience from the past industrial revolutions.

Simply put, AI looks to simulate human thought process and functioning on machines. The idea has been intriguing, yet fear-inducing in several ways to humans who fear that their existence would not amount to much if a machine could serve their purpose. Further, the technology has been advancing at a fast pace since 2012, a year that witnessed several breakthrough events in deep learning ( House, 2019). The years that followed had witnessed the technology rapidly integrated into aspects ranging from national security to day-today lives of individuals with its disruptive effects receiving wide coverage in all kinds of media. The final factor that drives the interest in AI is rooted in historical experiences. The coming of new technologies in the past have often turned existing power structures on their heads, a phenomenon that particularly stands true while speaking of industrial revolutions of the past. AI is an enabling technology like the steam engine or electricity and can boost the efficiency of anything that it is applied to ( Lyu, 2020). Actors who make prudent and effective use of the potential AI offers are arguably well-positioned to draw benefits to improve their relative position compared to their competitors and influence others’ behaviour to benefit themselves. In this context, a deep look is warranted into the ways in which AI can enhance the power status of a country. An examination into understanding the exact role that AI will play in shaping the outcome of major power competition in the current context is also warranted.

In pursuit of global domination or a relative improvement in their status, major powers including the USA, China, Russia, India, and the European Union (EU) have been seen to be ramping up their investments on AI research and development (R&D) to arrive at major breakthroughs. Several scholars argue that these nation-states are engaged in a ‘global AI race’ to utilise the technology to boost their economic productivity as well as military effectiveness to get ahead of the rest ( Geist, 2016; Savage, 2020; Levy, 2021; Stanford University, 2021). On the commercial front, AI can trigger largescale unemployment as well as enable the rise of new commercial technology giants. On the flipside, AI is said to have the ability to equip a country with formidable economic power by boosting its economic productivity ( Stevenson, 2018; Daniels & Chang, 2021). The potential for competition to achieve a technological breakthrough in AI is particularly said to manifest as an accelerated arms race that will create instability at national and international levels. This is because AI, like any other novel advances in technology can potentially facilitate newer forms and modes of warfare entailing highly advanced offensive and defensive capabilities wielded by the warring nation-states ( Araya, 2022; International Committee of the Red Cross, 2023). Increased integration of autonomous or semi-autonomous weaponry is further expected to increase the speed of warfare. In a projection for a worst-case scenario, AI and its contribution to reducing the human cost of war can possibly incentivise nation-states to engage in them frequently.

A number of publications in international relations, security studies and strategic studies have sought to study the effects of AI on warfare, global security and strategic stability ( Sweijs, 2018; Horowitz et al., 2018; Johnson, 2019; Chen, 2020; Ploumis, 2022). A scholarly discourse on contexualising the implications of AI within conceptual frameworks such as balance of power and power transition has also been evolving in recent times ( Wright n.d.; Lee 2018; Horowitz, Pindyck & Mahoney, 2022). While this discourse underlines AI as a central resource of great power competition, the larger impact of the technology on the overall distribution of capabilities and the structure of the international system have not been clearly addressed ( Ding, 2021; Granados & Pena, 2021). Moreover, the evolving discourse on AI in IR and security studies appear to largely overlook the nature of AI as a unique sociotechnical system. In addition to the technology itself, the impact of AI on the emerging world order will also be shaped by institutional and social dimensions along with artificial human-like entities ( Franssen, 2015; van de Poel, 2020). In this background, it becomes pertinent to try to understand how technological change alters extant power structures to shape the emerging global order. This article has been built on a foundation which contextualises the role of technology as a systemic factor in periodically re-ordering power hierarchies in the international system. It grounds itself on the understanding on national power and its systemic implications as understood through the lens of the balance of power concept in IR theory.

Exact predictions in this regard are impossible given the uncertainty surrounding the nature of evolution of AI in the current context. This article links theoretical forumalations with historical analyses, while placing the concept, nature and applications of AI within the same. Patterns of rise and fall of great powers in the international system following the First, Second and Third Industrial Revolution have been studied to delineate how diffusion of technologies alters relative power capabilities. The paper further utilises the concept of first mover advantage in technological innovation while placing it within the notion of great power status in IR. The study examines the impact of the advent of nuclear and space technologies to infer how a nation-state’s technological capabilities determine its power status. The qualitative analysis on the aforementioned aspects has been utilized as a basis to understand the extent to which AI can re-order international power hierarchies.

Key deductions have been employed to locate the relative standing of the USA, Russia and China through analysing their relative approaches and capabilities. The three nation-states are noted to have been entangled in competitive power dynamics amid ‘the ‘New Era of Great Power Competition’ ( Congressional Research Service, 2022) as China and Russia are increasingly seen to be challenging the US-led world order ( Allison, 2020; Savoy, 2022; Tiezzi, 2022). The three countries have consequently been identified as countries with stated intention to emerge as victors in the global AI race ( Simonite, 2017; Minevich, 2017; Lant, 2017; Garcia, 2019). Jeffrey Ding defines national success with respect to General Purpose Technologies such as AI as determined by “a state’s success in adopting them across a wide range of economic sectors” ( Ding, 2021, 3). The results of a state’s efforts to adopt as well as adapt to new technologies may potentially be conditioned by the nature of its domestic polity and socio-economic organisation. In this respect, the article tries to identify variables pertaining to the nature of domestic polity and socio-economic organization in the USA, China, and Russia that might determine whether these nation-states could draw from AI to enhance their power status in the international system. An attempt is finally made through scenario building to demonstrate a possible interplay between these attributes within the evolving geopolitical context.

## Technology as a catalyst of systemic change

The rise and fall of great powers in the modern era has arguably followed a techno-economic logic. Small Powers or Middle Powers might look to acquire technology to boost their National Power to rise to a Major power or Great Power status. Meanwhile, Power Transition theories in IR highlight a country’s ability to innovate as well as dominate in leading sectors as an important indicator of a country’s ability to rise to as well as preserve a superior power status. A country’s position in the international power hierarchy in this context is determined by whether a country can sustain its power through efficiently managing its technological growth. As well-acknowledged in the dominant discourse in IR, the periodic rise and fall of Great Powers in the international system has often occurred amid an overall milieu defined by the coming of new technological innovations. Countries may move up or down the power hierarchy based on how a country adopts and manages the disruption from a new technological innovation. Kennedy underlines this fact while explaining the rise and fall of powers as a consequence of change in relative military and economic capabilities as;

“differentials in growth rates and technological change, leading to shifts in the global economic balances, which in turn gradually impinge upon the political and military balances *(* *Kennedy,* 1989 *)* *.”*

The expectations on how AI will impact international relations in the coming years are largely based on these patterns as seen from time immemorial. For instance, the coming of chariots in the 1700 BC altered the power structures and changed the character of warfare in the ancient civilisations of Mesopotamia, India, and China and is said to have facilitated the movement of Aryans from Central Asia into Northern India ( World Supporter, 2014). Those in possession of the technology enjoyed a superior social standing which flowed from their ability to use the technology as a tool to subjugate the weaker classes. The revolution in iron smelting technology that came forth around the 1200 BC had a similar effect. With the availability of iron for armour and weaponry, infantry was effectively able to quickly neutralise chariots. The invention of stirrups in medieval Europe if further said to have paved the way for a power structure that would manifest in the form of Feudalism ( Derby, 2001). The use of stirrups by the Mongols is further said to have enabled their relentless, quick forward movement into invading territories ( Ingliss-Arkell, 2017). This is because stirrups would allow mounted knights to emerge as a core strike element of any major armed conflict of the time. Their status that would pervade into other aspects of social life to yield an unequal relationship between peasants and nobles in the society. The major centres of power in the modern and immediate pre-modern era such as the Ottoman, Mughal, Safavid, Ming, and Tokugawa empires rose to prominence through possession of superior strategic technologies, prominently gunpowder and cannons. Nicknamed as the ‘gunpowder empires’, these powers were able to subjugate their rivals and consolidate territory by utilising this technology against their awestruck rivals, although they were greater in number in several cases ( Kennedy, 1987).

This effect of technology on power dynamics between nation-states is seen to be much more profound in the Westphalian era. The three Industrial Revolutions that the world has witnessed over the past three centuries have altered the nature of the global order through causing the fall of prevailing great powers and facilitating the rise of others to occupy those positions ( Ding, 2021). The First Industrial Revolution which came about in the 18th century saw technologies including the steam engine revolutionise as well as boost the means of production. The United Kingdom (UK), the country that played host to these innovations reached the peak of its hegemony in the phase that immediately followed. The UK was able to defeat resistant inhabitants of its colonial territories in Asia and Africa who unsuccessfully employed inferior technology to attempt to defeat them and thereby consolidate their power over vast swathes of territory that held abundant resources.

Additionally, the spurt in manufacturing and mercantile friendly policies enabled it to become the economic epicentre of the world, its mastery over shipbuilding and naval technology bolstered its command over the seas and sea commerce, in turn allowing it to emerge as a formidable military power. In other words, the UK was successfully able to leverage its first mover advantage to emerge as a global hegemon reigning over the international system for at least two centuries that came after the First Industrial Revolution. First mover advantage is a term that finds its origin in marketing strategy. The term has been adapted to international relations to refer to an upper hand that a nation-state holds relative to others by being the first or earliest to develop or deploy certain technologies. A first mover advantage effectively allows a country to wield a “monopolistic control over certain innovations” and draw “significant advantages from it ( Gilli & Gilli, 2014, p.514). The term has been commonplace in discussions on military strategy and the geopolitical competition over technology in recent times. The notion of first mover advantage in the military context has been categorized into four: “first to innovate and invest, first to reveal, first to maneuver and first to employ”. The categorization reflects the level of maturity of the technology and its readiness to be deployed and the specific advantages that could be derived at each stage ( McClintock, Langeland & Spirtas, 2023, 2).

The era following the Second Industrial Revolution witnessed innovations including electricity and a boom in oil mining technologies occur in the USA. Meanwhile, Germany and Japan were able to advance in the chemical and iron and steel sectors. As these countries started accumulating capabilities through harnessing technological innovation, there was a minor upset over the prevailing balance of power. These countries were effectively able to utilise these technologies to improve their economic and military capabilities. The technology-power interplay is perhaps most visible in the case of Germany, following its reunification in 1871. Under the Prussian Empire, the country had forged ahead with its booming coal, iron, and steel and chemical industries ( Chandler Jr., 1990, 251). Along with facilitating a rapid development of rail networks ideal for troop movement, Germany would successfully translate its technological strength into rendering its military into a force that could defeat any other formidable power in the world. The massive arms build-up by other European powers in response to Germany’s amassing of techno-military might would eventually culminate in World War I. While Germany failed to translate its superior technological capabilities into a military victory, Germany’s technological rise and its bid to challenge the status quo would leave the UK significantly weakened. Moreover, this would further allow the USA and Japan to gain international influence as British hegemony was experiencing a relative decline.

British hegemony would ultimately come to an end by the conclusion of World War II with the USA donning the mantle of superpower in the decades that followed ( Lozada, 2005). The destructive might it demonstrated through using the atomic bomb in Hiroshima and Nagasaki in 1945 would go on to establish its status as an indisputable great power. By being the first to acquire this new weapon, the USA signalled the start of the decline of the UK’s supremacy, and also the beginning of a new power-balancing structure in world affairs. The USA’s acquisition of German blueprints for strategic technologies, prominently the V1 and V2, the earliest prototypes of the ballistic and cruise missile respectively, also significantly allowed the country to amass capabilities in a major way ( Jacobsen, 2014). It can be argued that the fundamentals gained from German technology is what enables the USA’s global force projection through various land, sea, air and space assets in the current context.

In spite of all the myriad political, economic, and structural changes that have occurred since 1945, the USA has continued to draw from its first mover advantage in nuclear weapon technology to preserve its position as a world leader, giving it the ability to set international norms in its interests. In addition to catapulting the USA to the superpower status, nuclear technology acted as one of the forces driving bipolar politics during the Cold War era, yielding both cooperative and competitive dynamics between the two blocs spearheaded by the USA and the Soviet Union. The five *de jure* Nuclear Weapon States (NWS) - the USA, Russia (the former Soviet Union), the UK, France, and China identified as so under the Nuclear Non-Proliferation Treaty (NPT), 1970, have managed to bolster their exceptional status through preserving their roles as sole holders of permanent membership at the United Nations (UN) Security Council. Their first mover advantage has further been preserved through setting and solidifying norms including the acceptability of peaceful uses of nuclear technology, non-proliferation, and the nuclear taboo which reinforces the notion that use of nuclear weapons is akin to the destruction of mankind itself ( Ying, 2019). Their interests continue to be protected by a series, institutions such as export control regimes and treaty arrangements including the NPT and the United Nations Conference on Disarmament.

Newer dimensions of the technology-power interplay can be identified from the experiences of the Third Industrial Revolution. The advent of technologies including advanced electronics and microprocessors would effectively render Information and Communication Technologies (ICT) indispensable to how nation-states carry out governance along with strategic and economic activities in the modern day. The array of technologies falling under the umbrella term ICTs, particularly those connected to rapid advances in computers and semiconductors, ushered in an ‘epochal shift’ from mechanised systems of the industrial era to information-based systems ( Galambos, 2013, 2-4).

Through serving as a medium fostering high level of interdependence among nation-states, ICTs effectively ushered in globalisation and paved way for the multipolar global order that prevails today. The USA which pioneered these technologies since the 1950s and 60s sought to benefit from the ICT revolution which by the 1990s. However, it would briefly feel threatened by Japan during the last quarter of the 20 ^th^ Century as the country would forge ahead in key areas of high technology such as electronics and semiconductors.

Japan capitalised on technology transfer from the US through means such as licensed production and efficient manufacturing techniques to establish a lead in advanced electronics and information technology. Japan’s lead in these sectors encouraged several scholars to predict its rise to challenge the USA as the world’s leading industrial power ( Lohr, 2011; Gilpin, 1997; Ozawa, 1974; Ingersoll, 1985-86). Fears were rife that Japan would abandon its pacifist posturing to convert its economic might into military and disrupt the prevailing world order at the time. Time would eventually douse these fears as the growth of Japan’s export-oriented economy stagnated around the 1990s. The USA would maintain its first mover advantage in ICTs effectively adapting its ICT-enabled service industries to computerisation. Moreover, the USA’s lead would also draw from its ability to cultivate as well as attract a pool of talent that can advance its lead ( Ding, 2021). The country was effectively able to capitalise on an opportune unipolar moment to integrate ICT into its military and economic activities. The Third Industrial Revolution effectively helped the USA strengthen its pre-eminent position through maintaining and improving its global force projection. Meanwhile, other countries such as India and the Association of Southeast Asian Nations (ASEAN) countries took advantage of the cheap manufacturing costs of the technology and human capital to exploit the opportunities provided by an interdependent world to emerge as powerful economies.

As highlighted in the above discussion, new technologies often interact with existing power structures at the systemic level to completely transform them. Its effect on relative power capabilities may eventually result in a rise as well as fall of great powers. While prevailing great powers positions are relatively relegated, small, middle or major powers might move into higher rungs. Such re-ordering is in turn shaped by a range of intervening variables. The, geopolitical context in which a country attempts to adapt the technology to boost its power profile is a key variable in play. The rise of Britain following the First Industrial Revolution for instance during a 100-year period of stability. The country had not engaged in any major wars from 1815-1914 ( Kennedy, 1987).

Factors including the nature of polity or economy may also influence changes to the prevailing power hierarchy. Key insights in this regard may be drawn from the decline of the Soviet Union amid the Third Industrial Revolution. Centralised control, a characteristic feature of the Soviet socialist economy is said to have caused the Soviet Union to fall behind the USA in terms of computing capabilities. The Soviet regime was also averse to individuals owning personal computers due to fears of subversion ( Curtis, 2018). Moreover, the inherent characteristics of the Soviet model of economic organisation is further said to have made little room for market agility, entrepreneurship and innovation ( Joshi, 1999). The ability and willingness of the leadership to adapt to and manage the integration of the technology prudently into various sectors have a role to play as well. The leadership has to set clear goals and standards while aiming to synergise activities and institutions managing the technology in order to achieve an elevation in power status. Other seemingly determinative variables include the rate of diffusion of technology, access to the required resources, and openness to change ( Vijayakumar, 2023).

The process of diffusion of technologies may also lead to an erosion of a state’s power. This is attested to by the evolving discourse on space weaponisation and its implications for strategic stability. An early mover advantage in spacefaring technologies granted substantial credence to the USA and the Soviet Union’s superpower status during the Cold War era. However, the scenario changed markedly following the USA’s withdrawal from the Anti-Ballistic Missile Treaty in 2002. Major powers including Russia and China perceived the same as a pretext for the USA to weaponise outer space. China’s 2007 anti-satellite test is said to have been motivated by their need to establish a space deterrent capability in this context ( Vijayakumar and Krishnan, 2023). Consequently, the USA’s bid to bolster its superpower status through space dominance was negatively affected, especially as various adversarial nations, including China, Russia, Iran and North Korea actively resorted to the testing of counterspace systems in subsequent years. The space assets of the USA are often referred to as the Achilles heel of its armed forces due to its reliance on these systems for maintaining its global force posture ( Defense Intelligence Agency, 2023). Hence, adversarial states being able to destroy, degrade or disrupt the USA’s space systems architecture permanently or temporarily could provide tactical advantages in a conflict situation and limit the USA’s response speed and efficacy.

Security challenges that erode power may also emanate from widespread diffusion of technologies into the commercial sphere. For instance, unmanned aerial vehicles was a technology that significantly aided the USA attain its military objectives during the Global War on Terror. However, drones in recent times have become “an evolving weapon of choice for terrorist organisations” who employ it asymmetrically to overcome nation-state’s conventional superiority ( Shaw, 2022). Such effects of technological diffusion that erode nation-state’s power through aggravating security vulnerabilities have featured within the evolving discourse on AI and IR. In the long run, people’s reliance on AI is likely to lead to some existential threats ( McClure, 2017). Such as the unemployment fiasco, moral and ethical risks, and personal privacy concerns that are often mentioned by scholars in the literature ( Kak, 2018).

As evident from history, changes to balance of power occur when enabling technologies driving industrial revolutions strengthen the economic and military components of national power. The military dimensions of power and its diffusion herein are of particular significance to consider. Two factors prominently determine the systemic effects of the diffusion of military innovation: adoption requirements and the capacity of institutions to meet such requirements through optimising resources or implementing changes ( Gilli & Gilli, 2014).

The likely trajectory of evolution of AI seen in terms of its ability to spur innovations across multiple sectors relevant for national power may arguably shape the evolving balance of power during the Fourth Industrial Revolution. Key insights in this regard may be gained through examining in depth the fundamental characteristics, components and nature of the technology.

## AI: Concept, nature, and applications

AI is a technology that is still unfolding. Hence, there are inherent limitations to predicting the exact form the technology might evolve to assume in advanced stages. ‘There is no broad consensus on the specific meanings of terms such as AI, autonomy and automation’ ( Horowitz, 2018). However, AI is broadly regarded as representative of efforts to mimic human thought and action in a machine. can be understood as an umbrella term for smart technologies that are aware of and can learn from their environments. AI enables computers and other devices to ‘sense their environment, learn, respond and think on their own’ and gain autonomy. They rely on software algorithms to perform tasks that would otherwise require human intelligence ( Pricewaterhouse Coopers, 2017, p. 2). In the current stage of AI development, it can perform activities including recognition of patterns, statistics, and images and natural language processing. However, even in its routine tasks, such as image classification, AI in its current form is generally seen as unable to perform activities using common sense like humans, but instead relies on the ways that it perceives the inputs ( Scott, Heumann, & Lorenz, 2018).

There are two broad approaches in AI development: symbolic and connectionist. In symbolic AI, the algorithms operate through deducing key behavioural pathways, while the connectionist approach trains algorithms to solve problems through calculations. In this regard, algorithms learn to perform complex tasks through two prominent types of learning as described in programming parlance namely machine learning and deep learning. While machine learning uses computational techniques such as decision trees to feed information and rules to the algorithm, deep learning relies on neural networks that function much like a human or animal brain to think and perform tasks such as image recognition ( Dong, 2017).

In this background three types of AI can be identified: Artificial Narrow Intelligence (ANI), Artificial General Intelligence (AGI), and Artificial Super Intelligence (ASI) ( Fourtane, 2019). Much of AI that is in use today falls within the scope of ANI. In ANI, algorithms learn from a specific data set to acquire the capability to solve single, rather straightforward problems, such as identifying certain objects from images, monitor weather, or play chess. While it may appear that narrow AI is capable of quick-thinking to understand complex scenarios, the scope of the technology is narrowly pre-determined. The next stage of evolution of technology is AGI wherein the algorithm can mimic an average human brain in performing tasks of varying levels of complexity. ASI has been said to be the ultimate form of evolution and represents the point where AI surpasses human intelligence to become super-intelligent machines, the entities that alarmists fear would bring about the elimination of the human race. According to a scientific school of thought the achievement of ASI will trigger the stage of singularity which would result in ‘unforeseeable, irreversible changes to human civilisation’. According to a version of this theory, the super-intelligent entity will keep upgrading itself hence causing an ‘intelligence explosion’ ( Hvistendahl, 2019).

As previously noted, much of AI in use today is ANI with the technology being trained on a specific dataset to perform specific tasks such as data or image processing, identification of patterns, and so on. In addition to speeding up processes that humans take time to perform such as sorting through and connecting millions of data points, combinations of AI and robotics can also be used to perform tasks that are considered too risky or dangerous for humans. Such applications could become more widespread in scenarios such as disaster relief and rescue as well as in military applications such as detection of mines or Improvised Explosive Devices (IED) or guarding of borders. For instance, the USA has been using TALON, a military robot for identifying and disposing IEDs in locations such as Bosnia and Herzegovina, Afghanistan, and Iraq in the post-1990s era ( TALON Tracked Military Robot, 2020). The USA’s Army is further said to have deployed the Special Weapons Observation Remote Reconnaissance Direct Action System (SWORDS), an improved variant of TALON fitted with a semi-autonomous gun in Iraq ( Wired, 2007). Objections and ethical concerns have been raised against Lethal Autonomous Weapon Systems (LAWS) that can potentially strike targets without human oversight by prominent figures including Stephen Hawking and Tesla and SpaceX CEO Elon Musk ( Cifford, 2017). While these weapons are perceived by critics as killer robots with their own ability to kill, nation-states including the USA and China are not looking to remove human oversight upon LAWS. However, there are nuanced differences with respect to their individual perceptions on what constitutes meaningful human control. As evident from its position as articulated at the United National Group of Governmental Experts (GGE) in 2017, China has put forth a narrow understanding on LAWS ( Qiao-Franco & Bode, 2023, 107). As per China’s understanding, LAWS are characterised as per five essential features ( Group of Governmental Experts 2018):

i) Lethality, which implies that a weapon must carry enough firepower for it to be considered lethal.
ii) Autonomy, signifying a lack of human intervention during the system’s functioning.
iii) Impossibility of termination, which refers to the ability to stop or terminate the system once activated.
iv) Indiscriminate effects, pertain to the system’s ability to perform its function irrespective of specific scenarios or targets.
v) Evolution refers to the system’s ability to learn from the environment and evolve to expand its capabilities in a manner that exceeds human expectations.

Meanwhile, the USA understands LAWS as “weapon systems that, once activated can select and engage targets without further intervention by an operator” ( Department of Defense 2023, 21). This understanding encompasses those systems that can select as well as strike targets in the absence of operator input after activation as well as those in which the operator can override the system. Moreover, this understanding seemingly limits human control to the activation phase, while remaining ambiguous on the operator’s ability to override. The scope of human supervision may face several challenges if and when the technology advances to a level beyond human comprehension ( Scharre, 2018).

While AI offers lucrative options to militaries, the technology is pervading rapidly into various sectors of the economy to become an omnipresent part of how products and services are developed, manufactured, and sold. Such uses of AI-enabled predictive analytics seem to be growing in sectors ranging from healthcare to outer space. Combinations of ANI with big data analytics is already being tried out in various arenas such as in the development of models to analyse the spread of the COVID-19 pandemic, to assess the nature of the disease as well as the patterns of its spread. Use of algorithms has also become a quintessential element of modern-day marketing with AI learning from user’s data to gauge their preferences and offer suitable options for them. AI, big data analytics, and robotics is further expected to improve the quality and quantity of products and speed up as well as streamline manufacturing and movement of goods and services hence boosting the overall economic productivity of a nation-state.

The characteristics of AI are markedly different from the enabling technologies that paved the way for the three industrial revolutions of the past. Unlike the internal combustion engine, electricity or ICTs, the efficiency in the context of AI is seen as an effect of its ability to mimic or even overcome quintessentially human attributes, including logical thinking, sensing of the environment and emotions. This magnifies the level of unpredictability surrounding the technology and its purported implications on the emerging world order. The achievement of ASI as well as the widespread diffusion of machine intelligence in particular can potentially alter the anarchic nature of the world order. As per the Singleton Hypothesis proposed by Nick Bostrom, all intelligent life on Earth (including human and artificial) will eventually evolve to become a single entity termed the singleton ( Bostrom, 2005). This entity shall be akin to a world government and shall control all aspects of the global society including security ( Zweibelson, 2023).

While the singleton hypothesis paints a futuristic scenario, existing narrow AI has been noted to several issues including bias and the blackbox problem which restricts human ability to understand how a system arrived at a particular decision ( Vijayakumar, 2023). Integrating these systems into high stakes decisionmaking without remedying these inherent issues may negatively affect national power. The emerging world order in this context may be shaped by how institutions in nation-states prioritise or guide the development of AI in the military and commercial domains. Such priorities may be developed in light of a nation-state’s unique geopolitical and security circumstances and resource constraints. To get ahead in the AI race through military or economic means, the contenders may place an equal amount of emphasis on both with both the strands building into each other. The next section highlights the key pillars of AI plans of major powers.

To get ahead in the AI race through military or economic means, the contenders may place an equal amount of emphasis on both with both the strands building into each other. Nation-state’s priorities with respect to AI may further be shaped by their unique geopolitical and security circumstances. The next section highlights the key pillars of AI plans of major powers.

## AI strategies of major powers: An overview

Given the hype surrounding AI, the central contenders of the global AI race namely, the USA, Russia and China, have started to place a large amount of importance on it. The governments of these countries have further been engaged in charting out legal and policy frameworks to facilitate faster development of AI, to reap its full potential as well as manage the disruption that it already seems to be bringing about. A glance at the national AI strategies of the USA, China, and Russia provides key insights into where the powers currently stand relatively to each other.

### China

China recognises AI as a strategic technology that will guide international competition in the future and underlines its role in the protection of national security as well as in enhancing national competitiveness. As per China’s New Development Artificial Intelligence Plan released in July 2017 ( DigiChina, 2017), it has its eyes set on becoming a world leader in AI by 2025. Meanwhile, the Made in China 2025 document reflects China’s intent to employ Fourth Industrial Revolution technologies including AI to implement an industrial transformation. In doing so, China seeks to emerge as a global hub for high-tech manufacturing ( McBride and Chatzky, 2019). In doing this, it is looking to challenge the USA’s supremacy in both the civilian and military spheres. Beijing here is following the doctrine of ‘civil-military fusion’ which entails a blurring of lines between civilian and military resources in pursuing advances in science and technology ( Tay, 2020). The goal here is to channel all national energies including those of academic institutions, military and private players to fasten the country’s military modernisation as well as economic growth ( Pecotic, 2019). In the military sphere, AI is central to the People’s Liberation Army’s (PLA) ‘intelligentization doctrine’ ( Bassler & Noon, 2022). This accords for the technology an important role in helping commanders with strategic decision-making through analysing volumes of data including satellite imagery and GPS locations of troops that they are expected to manoeuvre.

The government has further hand-picked Chinese AI giants, Tencent, Alibaba, Baidu, and iFlytek (a leading speech recognition company) as its dream team, tasking them to work on different priority areas ( Jiang & Dai, 2017). While Baidu has been tasked with technologies relating to autonomous driving, Tencent has been tasked with looking at AI in healthcare and medical diagnostics. Alibaba Cloud (Aliyun) is looking into smart cities. Meanwhile, China has also been integrating AI into its domestic governance to maintain stability and exercise social control ( Ding, 2018). Rampant surveillance using facial recognition cameras are used to identify those who violate traffic rules and commit crimes. It’s policy in the Xinjiang Autonomous Region using AI algorithms is specifically intended at racial profiling to track and control the Uighurs ( Mozur, 2019; Wakefield, 2021).

Chinese AI policies from 2019 are said to have “articulately” incorporated elements of AI ethics ( Qiao-Franco and Zhu, 2022, 8). The New Generation AI Governance Principles-Developing Responsible AI document put together by an expert committee constituted by the Ministry of Science and Technology highlights eight principles guiding the “safe, controllable and responsible use of AI. These include harmony and friendliness, fairness and justice; inclusiveness and sharing, respect for privacy, security and controllability, shared responsibility, open cooperation and agile governance” ( Library of Congress, 2019). The ethical principles outlined in the 2019 guidelines have been incorporated into legislations including the Data Security Law and the Personal Information Protection Law enacted in 2021. While the former emphasises on compliance to laws and ethics in data collection, the latter stresses on the notion of the users’ informed consent in data collection. However, the government can disregard such ethics and responsibility in grave situation that may be deemed as a threat to national security or public interest.

### The USA

The USA’s Department of Defense’s (DoD) Third Offset Strategy released in 2014 places a heavy emphasis on AI to be integrated into the military domain to maintain a military edge over near-peer competitors, Russia and China ( Gentile, *et al.*, 2021). Moreover, through this document, the DoD has discussed how the USA can employ technology to negate its military disadvantage *vis-a-vis* its competitors, placing a heavy emphasis on human-machine collaboration. This has been termed the ‘Centaur Model’ and aims to combine the efficiency of humans and machines in a way that they complement each other by addressing each other’s flaws ( Horowitz, 2018). The Pentagon’s AI strategy released in February 2019 ( US Department of Defense, 2019) further laid out specific priorities in this regard. It states that AI will be integrated in areas including ‘improving situational awareness and decision-making, increasing the safety of operating equipment, implementing predictive maintenance and supply, and streamlining business processes’ ( US Department of Defense, 2019, 7). In doing all this, the overall goal is to augment the capabilities of the troops to free them of “tedious, cognitive physical tasks” ( US Department of Defense, 2019, 7). The emphasis here seems to be on handing over to AI the tasks that humans may find mundane and time-consuming such as analysis of surveillance data ( Cassano, 2018). For instance, the DoD’s Project Maven - which witnessed a 580% increase in funding from 2018 to 2019 - is intended to analyse feeds collected by thousands of drones ( Cassano, 2018). Meanwhile, robots are deployed in the field to perform dangerous missions such as detecting IEDs. On the commercial front, leading players including Facebook, Apple, Amazon, Netflix, and Google (commonly referred to using the acronym FAANG), as well as Microsoft, have an edge over Chinese firms in access to data owing to their collective dominance over the global technology landscape ( Mulrenan, 2020), which is crucial for AI’s evolution.

### Russia

Compared to the USA and China, Russia is often not regarded as a “frontrunner in the global AI race” ( Nair, 2022) and has been termed akin to an outsider in the same ( Nacetti, 2020). Russia lags significantly behind China and the USA in terms of key indicators that are employed to assess a country’s technological capability such as number of patents, publications in journals and total investment in R&D. The strength of its digital economy has been deemed weak with fewer private players involved. While Russia is said to have a strong basis in mathematics and basic sciences ( Pecotic, 2019), it is said to have shortcomings in terms of talent that can be employed in developing AI ( Petrella, Miller, & Cooper, 2021). It is further affected by the ‘brain drain’ phenomenon with prospective talent often migrating to the USA or Israel in search of lucrative opportunities. In a stark contrast to China and the USA, Russia further appears to rely on state-owned entities to innovate as well as implement its AI strategy ( Petrella, Miller, & Cooper 2021). Much like China, Russia handed over the task of developing roadmaps for developing technology to various state-owned enterprises. While Rostec was tasked with the 5G implementation roadmap, Rosatom was assigned the task of developing a quantum computing roadmap. Other state-owned entities such as Sberbank, Rostec, Yandex, and Gazprom Neft have been tasked with developing AI systems for their own diverse purposes, such as improving bank operations, streamlining military manufacturing, creating driverless cars and managing oil production, respectively ( Petrella, Miller, & Cooper 2021, 81).

Russia’s 2019 AI strategy ( Ministry of Digital Developments Communications and Mass Media of the Russian Federation, 2019; Bendett, 2019) sets out a goal for the country to become a leading contender in the global AI race through sharpening its existing capabilities in science, engineering and mathematics and through making coding expertise available. It also emphasises upon AI ethics and data sovereignty by calling for various kinds of data including those from surveillance systems, weather, sound, and medical sources to be stored in Russian databases. It further lays focus on legal and ethical aspects with respect to the handling of data as well as to govern the “interaction of the individual with AI” ( Bendett, 2019). It further emphasises upon integrating the technology in the healthcare and education sectors. The role of the private sector here seems relatively muted in comparison to the USA and China. However, Russia is rather reticent on its intentions toward military ( Bendett, 2019) applications of AI although it has been forging ahead with plans for using AI to strengthen its military. Russia’s intentions for military applications are largely unknown, although it has already been using the technology to make smarter weaponry. Russia’s strategy for AI in the military domain rests on two key pillars: the strengthening of its existing weapons and platforms through integrating AI as well as through use of AI in asymmetric tactics. Russia is believed to be integrating AI into weapon systems such as those entailed in electronic warfare, air defence, guided missile systems, and drones.

It is reported to have tested several of these systems including the Uran-9 autonomous tank in the Syrian conflict ( Robitzski, 2019) where its military tactics also underwent several changes. These ambitions can also be seen reflected in Russia’s plans for modern systems including the Su-57 multi-role fifth generation fighter jet as well as the T14 Armata Main Battle Tank. Russia is also said to be working on a smart missile which can alter its course based on the incoming missile defence system ( Futurism, 2017). AI also occupies a central place in Russia’s hybrid warfare strategies. The country is said to use AI algorithms to power its disinformation campaigns intending to influence the politics of other countries ( Polyakova, 2018). Such tactics have been evident from its military campaigns in Ukraine in both 2014 and 2022 ( Kuzio, 2019; Baumann, 2020; Blankenship & Ordu, 2022).

The three countries appear to recognise the opportunities and challenges that AI presents and have been striving to strike a balance between power ambitions and the expected fallouts from disruption eroding power. Given the ubiquity of AI across multiple sectors relevant to military and economy, policy and institutional synergy at various levels including civilian and military, and between public and private can be identified as pivotal to draw dividends relevant for power. The presence of a strong private sector equips the USA and China with significant advantages, Russia may be capitalising on existing military strengths and hybrid warfare tactics to target the USA’s vulnerabilities and erode the credibility of its power through means such as disinformation campaigns. Meanwhile, the USA and Russia may have significant advantages as countries which have been actively engaging in various active conflicts around the world in recent times. This allows them to manage their expectations from the current level of maturity of the technology as well as better them based on practical experiences.

Diplomatic manoeuvring can also play a key role in helping these nation-states overcome their shortcomings and impact the evolving balance of power. The USA for instance has been building the collective AI capabilities of its alliance network through minilaterals such as the Quadrilateral Security Dialogue (Quad) and the AUKUS grouping. Its bilateral engagements with Taiwan and India have strived to build win-win arrangements that mutually boost their AI capabilities ( Hadda, 2021; Aisyah, 2021). Meanwhile, Russia and China have been jointly trying to balance against the USA’s efforts to pool power by strengthening their own strategic partnership. Under the terms of the Joint Statement between the People’s Republic of China and the Russian Federation on Deepening the Comprehensive Strategic Partnership of Coordination in the New Era” signed in March 2023, China and Russia look to combine their wealth of research capacity and industrial capabilities to emerge as world leaders in information technology, cybersecurity and artificial intelligence” ( Sharwood, 2023). In this way, the emerging global order, while characterised by multipolarity, increasingly seems to resemble Cold War era power blocs and can potentially move towards bipolarity in the coming decades ( Huasheng & Kortunov, 2020; Xuetong, 2023).

## AI and the emerging world order

While AI could usher in a range of technological innovations, these need not always translate to military innovations that demand radical changes in organisational characteristics or war-fighting strategies. Since AI technology is relatively nascent at this stage and given the unpredictability surrounding its applications, it is far too early to draw comparisons with previous instances of major technological change, such as that of the development of tanks during World War I. In this context, the purpose is best served by examining the capabilities major powers while placing them within the organisational attributes that are most likely to position entities that constitute a nation-state such as the government and the military to adapt quickest to a major technological change and achieve an edge.

The Adoption Capacity Theory posits that specific organisational and financial considerations of militaries determine the rate of diffusion of technological innovations and its impact on the balance of power. While financial considerations relate to attributes including the cost per unit for hardware and other investments, organisational considerations pertain to the military mindset, specifically as to how the military would perceive the resultant changes in war-fighting, or existing bureaucratic practices that may obstruct certain actors from facilitating the adoption of technology ( Gilli & Gilli, 2014). As discussed before, technological innovations by themselves do not impact the balance of power, although the way in which the technology is used may set a military apart ( Posen, 1984).

For instance, the British Royal Navy invented the aircraft carrier to operate as a platform for surveillance aircraft to spot battleships. Invested with a traditional mentality, the Royal Navy saw carriers as an aide to battleships. However, rising naval powers, Japan and the USA were quick to realise that the most effective use of the aircraft carrier was as a mobile attacking platform. In this case, the UK found itself at a disadvantage in terms of the mindset, as it would have been difficult for commanders to think outside the box. The emerging navies of USA and Japan were less bogged down by such considerations. Therefore, the chances are that countries with powerful battle-hardened militaries might find themselves at a disadvantage when it comes to adopting AI, while emerging powers could hold the advantage.

As elaborated in the above discussion, the scale at which AI can shape the global balance of power depends on three factors: firstly, the rate of development and diffusion of technology, secondly, the mode of utilisation and level of institutionalisation, and thirdly, on domestic factors. Potential changes to the balance of power may result from patterns of diffusion that may follow the achievement of a breakthrough in AI such as that of AGI. The rate of diffusion of technology is crucial to consider in this context. Whether an AI breakthrough results in the rise of a hegemon will depend on whether the state which pioneered the breakthrough is able to sustain the first mover advantage through various means. Whether the breakthrough innovation occurs in military or the civilian sphere may determine such patterns. This point may be best illustrated by understanding the patterns of diffusion that nuclear technology followed in the post-World War II era. Breakthroughs in nuclear sciences such as the nuclear fission in the civilian sphere during the 20 ^th^ Century paved the way forward for the development of various technologies and applications. However, it was military research & development such as the nuclear bomb and its delivery systems, which proved to be impactful breakthroughs which continue to shape global power dynamics to the present day. After the hierarchical NPT structure came into being in 1968, nuclear technologies have diffused under strict monitoring and verification stipulations. This has undoubtedly increased the difficulty for any non-NWS to progress to the level of acquiring the nuclear weapon. Except for the NWS, merely three states, namely, India, Pakistan and North Korea have tested nuclear weapons in the post-Cold War era. The NWS were essentially able to use their exceptional status and secure their collective first mover advantage to sustain a viable strategic upper hand on international affairs.

ICT in contrast represents a widely commercialised military innovation. Low costs of manufacturing and ease of imitating within a globalised geopolitical context enabled several countries to rise to become major poles of the international system. Meanwhile, a technology such as the Global Positioning System (GPS) also diffused from the military to the civilian domain. The development of the GPS started in 1974 when the U.S. Air Force started work on the very first fleet of Navstar satellites. The Ronald Reagan administration in 1983 adopted a policy to make GPS available for civilian users, free of cost. Once the US government itself triggered the technology diffusion, handheld units equipped with GPS were available in the market as early as 1989 ( Aerospace Corporation, 2023). In order to address the threat from the adversaries using the GPS, the Selective Availability (SA) function was included within the NAVSTAR satellites. SA worked to degrade the accuracy of the system “by a factor of 10” for civilian users through deliberately introducing errors into satellite data ( Martin and Bastide, 2015, 613).

The first mover advantage is hence likely to wither away once AI applications become imitable. While it can be argued the AI applications exclusively meant for the military are difficult to mimic, private industries as drivers of AI technology innovation might change the tide. Particularly, the nature of the relationship between governments and industries will provide insights into how fast nation-states can follow the first mover. Whether military innovations diffuse to the commercial domain itself may be determined by the prevailing traditions guiding public-private synergy in a country. Schmid identifies three pathways through which defense expenditure shapes the trends in innovation in the civilian sphere. Knowledge diffusion to this end can occur through defence institutions engaging institutions including universities, research centres and private companies. For instance, in the USA, the tradition that has prevailed since the Cold War years is said to have helped create a university-based research infrastructure. This infrastructure has been able to serve as an important source of civilian innovations and manpower in the subsequent decades. Defence spending may also drive civilian information through increasing demand and competition as companies who seek to win government contracts may ramp up their own R&D. Empirical evidence in this regard has existed since the 18th century. Defence R&D can thirdly drive civilian innovation through production of technologies of an enabling nature that can enhance subsequent innovative outputs ( Schmid, 2017).

Both the USA and China are heavily relying on private players to develop AI technology. Dual-use technologies that private players are developing, such as facial recognition, could form the basis of potential applications in national security. Existing in the world’s most technologically advanced nation, American technology leaders including Google, Microsoft, and Facebook are favourably positioned to operate in a nurturing business ecosystem, characterised by less government oversight. However, increasing voices arguing for more government regulation of technology, particularly in the cyber domain, pose a challenge to relations between the government and the private sector, and could prevent ambitious partnerships due to a lack of trust ( Huddleston Jr., 2019).

Meanwhile, in China’s authoritarian capitalist economy, tech giants including Alibaba and Tencent maintain close ties with the government, whereas the business ecosystem thrives despite strict regulations. China’s AI strategy seems to follow a larger governmental outline, with designated roles for various actors including the industry and academia. Russia is at a relative disadvantage given its relative weaknesses in the digital sector. Unlike China and the USA, Russia has no significant private players to join hands with. This could perhaps be the reason why Russia is focusing on using AI to better its military strengths and through asymmetric means. Instead, Russia’s defence industry, which works closely with the Kremlin, is spearheading research on AI in the country. Recent technological advancements Russia has showcased, such as the Avangard hypersonic missile, clearly shows the resurgent capabilities of this Russian military-industrial complex ( Garcia, 2019).

While the USA remains the world’s most technologically advanced nation, China is fast catching up and envisions utilising AI and robotics among other things to level the playing field with the former. For China to replace the USA as the most advanced nation in the world, it is safe to say that it needs to come out clearly on top in the AI arms race. In keeping with the predicted scenario where the ones who master AI become masters of the world, China would have used its advantages in AI to attain parity with the USA. What could make this scenario possible is the ability of China’s ruling dispensation to maintain the level of control it has over all the public and private machineries functioning in its state and ensure dissent to the introduction of AI remains low-key. Indeed, through other policies aimed at opening up the markets it has access to, China is showing an awareness of ensuring AI does not take away jobs from its large population, and instead can open more opportunities for an upwardly mobile citizenry. Moreover, the USA’s military as an older, more experienced organisation, may have to cross multiple bureaucratic obstacles to adopt AI into their organisation. While it is next to impossible to say who will achieve a definitive lead, both nations will benefit from being at the forefront of the AI race and will inspire similar research to follow in other nations.

Eventually, the attaining of a Sino-American parity on AI might proceed on similar lines to the evolution of nuclear technology during the middle of the Cold War. Keeping strategic gains in sight, both countries may share technology with friendly nations through trade or partnerships, with some information leaking out through channels such as espionage. Particularly if information about commercial AI remains open source, these nations aspiring for a place at the high table of global affairs will be able to follow in the footsteps of China, the USA, and Russia, which based on current indications should continue to be a leading player in the race. The three powers may also develop countermeasures to defend against and anticipate adversarial operations, leading to a multiplication of technological applications such as confusing AI through manipulating images or triggering illogical reactions. War strategies are also likely to evolve to include a range of asymmetric tactics intended to stave off an adversary’s superior AI technology. However, the possibility of attaining a broader parity in terms of military capabilities is highly unlikely or in any case, could take a long time.

## Conclusion

In pursuit of global domination or a relative improvement in their status, major powers including the USA, China and Russia, have been ramping up their investments on AI R&D to arrive at major breakthroughs. Several scholars argue that these nation-states are engaged in a ‘global AI race’ to utilise the technology to boost their economic productivity as well as military effectiveness to get ahead of the rest. For the USA, the prevailing superpower of the international system, AI offers the means to preserve or strengthen their existing position. In other words, AI can arguably compensate for the dent in its international perception due to a perceived decline of power following its misadventures in Afghanistan and the weaning away of economic dominance to China. Meanwhile, the latter seemingly views AI as a shot in the arm that can boost its national power to either supplant the USA or at least match its might to serve its own ambition to emerge as a global superpower in the next few decades. Russia’s perspective partially reflects the Chinese view with the country looking to employ AI in combination with asymmetric cyber tactics and space weapon technologies to enhance its existing military capabilities to gain an edge over the USA. Through AI, Russia hopes to get the USA to the negotiating table through which the country hopes to regain its past glory. The evolving trajectory of the global AI race and its manifestations on relative power capabilities can have a significant impact on the emerging world order.

This study has drawn from historical experience to assess whether major powers in the international system today, the USA, China and Russia can harness the opportunities offered by AI to enhance their power status as well as goals envisioned under national interest. Each of these players have variations in their approaches as well as differing abilities as organisations to absorb the technology into the fundamental ways in which they operate. However, possession of advanced capabilities does not by itself guarantee power as an outcome or the elevation and subsequent sustainment of a superior power status. In the oft-quoted words of Russian President Vladimir Putin “AI comes with colossal opportunities, but also threats that are difficult to predict. Whoever becomes the leader in this sphere will become the ruler of the world.” ( Vincent, 2017). As evidenced by the history of technological change, a country can only rise to a superior power status when its institutions operate in synergy to tackle challenges from technological disruption, while utilising the opportunities. Such policy synergy has to be guided clearly from leadership, which also has to show willingness to change existing policies as required, to facilitate the adoption of AI ( Vijayakumar, 2023; Milner and Solstad, 2021).

The nature of AI as an unique and novel kind of sociotechnical system including artificial human-like agents poses challenges to projecting its exact impact on the global order. A large part of whether the USA, China or Russia forge ahead and the ways in which the rise or fall these powers might come about is also contingent on whether the technology evolves from ANI to AGI or even ASI. Evolution to both AGI and/or ASI is likely to yield different projections given the geopolitical circumstances of the time. Such a breakthrough shall fundamentally alter the nature of the world order and of power as it is currently understood in IR. If economics is to shape the contours of the competition, China is likely to achieve its superpower ambitions and emerge as an even more powerful player in international affairs. The USA holds a unique advantage in terms of the wealth of technological knowledge it possesses as well as through its private ecosystem which is churning out AI innovations at an ever-increasing rate. The USA also has been consistently attracting the smartest minds from around the world, which can fuel its fast-paced development. The world system will likely continue in its multipolar configuration in this case with commercial AI diffusing fast to enable multiple poles of power to strengthen their position.

If military innovation and its impact on war decides the outcome of the AI race, the USA and China are well-positioned to emerge on top. While the USA still has a substantial lead in capabilities and fighting experience compared to the Chinese, AI might slightly change the equation in China’s favour. China’s authoritarian system of governance, access to data, and smart policies such a civil-military fusion may further put the country at an advantage. In this regard, a notable trend within the current multipolar environment points to the evolution of nascent technology-based power blocs, one led by the Sino-Russian alliance and the other represented by the USA and its alliance. Growing strategic competition among the two power blocs could lead to cause the world order to move to a bipolar configuration int the coming decades. Similar to how World War II terminated Germany’s rise trajectory, the power potential of any of the contenders discussed in the article may be constrained by the outbreak of a major war that they involve themselves in. Ultimately, the winner(s) of the AI race may be those who adopt a prudent approach to the technology and make calculated moves towards using this unique AI moment to rise to the top.

## Data availability

No data are associated with this article.
