# Supplementary material for: Potential impact of artificial intelligence on the emerging world order
Source: F1000Res. 2023 Oct 30;11:1186. Originally published 2022 Oct 17. [Version 2] doi: 10.12688/f1000research.124906.2 (PMC10618639; doi:10.12688/f1000research.124906.2)
Supplement: Peer review history

# Reviewer report: Reviewer response for version 2

**DOI:** 10.5256/f1000research.157578.r219317

**Published:** 2023-10-31

**Authors:** Guangyu Qiao-Franco

**Affiliations:** 1 Radboud University, Nijmegen, The Netherlands

The revised manuscript has addressed my concerns regarding the earlier version sufficiently. Good work on this!

Is the work clearly and accurately presented and does it cite the current literature?

Partly

If applicable, is the statistical analysis and its interpretation appropriate?

Yes

Are all the source data underlying the results available to ensure full reproducibility?

Yes

Is the study design appropriate and is the work technically sound?

Yes

Are the conclusions drawn adequately supported by the results?

Yes

Are sufficient details of methods and analysis provided to allow replication by others?

Partly

Reviewer Expertise:

Artificial Intelligence, Cyber Security, China Studies, Norm Diffusion, International Organisations.

I confirm that I have read this submission and believe that I have an appropriate level of expertise to confirm that it is of an acceptable scientific standard.

# Reviewer report: Reviewer response for version 1

**DOI:** 10.5256/f1000research.137148.r180402

**Published:** 2023-07-03

**Authors:** Guangyu Qiao-Franco

**Affiliations:** 1 Radboud University, Nijmegen, The Netherlands

This manuscript engages with the important topic of AI and geopolitics. Overall, it is a strong, well-written and researched paper. It contributes to the literature by situating AI competition in a broader historical context of technology evolution and setting out clearly the AI ambitions and development status of China, the US and Russia. I support its publication, and my recommendation for the current manuscript is for minor revisions that bring more focus and depth to the theoretical argument and empirical operationalisation. Below, I offer comments that will assist the author in improving the piece.

 First, I would suggest the author state her stance to the research question of whether major powers can harness the opportunities offered by AI to enhance their power status and shape the world order explicitly in the introduction. This will help clarify the contribution of the paper and the need for studying the subject matter of AI and international order. (The conclusion can also be stronger in order to highlight this important original contribution that the author has set forth.)

 Relatedly, the author can be clearer on what the specific contribution of the paper is and in what ways this research builds on previous contributions on this topic. The topic of AI and geopolitics has already been studied elsewhere, albeit to a limited extent (see e.g., Bode *et al.* 2023 ^1^ published in Ethics and Information Technology, which similarly compares the practices of China, the US and Russia). In addition, the issue has been extensively discussed at the UNGGE on LAWS. The author can do a more thorough job of situating the study in the existing literature(s) and teasing out the innovative points it brings to the table.

 Another issue is with the AI definition. The term AI is used a bit unproblematically throughout, as if its referents were self-evident. Since the term can be interpreted differently by readers, a clear definition of the term would be helpful.

 I would also encourage the author to consider adding some reflections in three empirical sections on how the power balance and international order will evolve in the light of these discussions. For instance, to what extent, can we expect the trajectories of technology development and power shift to continue in the Fourth Industrial Revolution in the context of AI? What do the different policy approaches to AI of China, Russia, and the US say about the IR landscape? This will be helpful in clarifying and substantiating the theoretical discussion developed from p. 9 onwards.

 The manuscript can also better source all the ideas it is drawing upon to better engage with the literature. The following statements all need to be sourced: “AI can equip a country with formidable economic power.” (p. 3); “The potential for competition to achieve a technological breakthrough in AI is particularly said to manifest as an accelerated arms race that will create instability at national and international levels. This is because AI can potentially facilitate newer forms of warfare entailing highly advanced offensive and defensive capabilities wielded by the warring nation-states.” (p. 3); the concept of “first-mover advantage” (p. 5); and “While it may cause the rise as well as fall of great powers, the geopolitical context as well as factors including the nature of polity or economy, the nature of leadership, and of the technology itself will determine whether the prevailing great powers can employ the technology to bolster their predominant status.” (p. 6). In absence of proper in-text citations, the analysis comes off as generalized (and unsubstantiated) personal opinion.

 Some other sentences and claims made by the authors need further clarification or refinement. These include, for instance, the following:

 p. 7 “While these weapons are perceived by critics as killer robots with their own ability to kill, nation-states including the USA and China are not looking to remove human oversight upon LAWS, although the scope of human supervision may face several challenges if and when the technology advances sufficiently for humans to comprehend its actions (Scharre, 2018).” This argument is largely true but can be refined. States such as China and the US agree on retaining human oversight over weapons systems but differ on what constitutes human control. The US for instance has a much broader understanding of human control (or human judgement) than many other countries, especially developing countries.

 pp. 7-8 discussions on China’s AI policies are not updated. Two of my latest papers might be helpful:

- Guangyu Qiao-Franco, Ingvild Bode, Weaponised Artificial Intelligence and Chinese Practices of Human–Machine Interaction ^2^

- Guangyu Qiao-Franco & Rongsheng Zhu (2022) China’s Artificial Intelligence Ethics: Policy Development in an Emergent Community of Practice ^3^

p. 8 “Russia’s intentions for military applications are largely unknown, although it has already been using the technology to make smarter weaponry.” I do not agree with this point as Russia’s position has been fairly clearly stated at the UNGGE on LAWS meetings. A few studies have been developed in this vein. An example would be Anna Nadibaidze (2022) Great power identity in Russia’s position on autonomous weapons systems ^4^.

 p. 9 “The first mover advantage is hence likely to wither away once AI applications become imitable”. This point can be developed by drawing on literature discussing the proliferation risk of AI weapons. For instance, Denise Garcia, Lethal Artificial Intelligence and Change: The Future of International Peace and Security ^5^.

 p. 10 “In comparison, the PLA seems more willing to take chances with giving AI wider scope and responsibilities and could stand to benefit from this.” I am not convinced of this point as the PLA similarly faces political restraints and takes into account the risk of AI to human control in AI development and deployment. See my paper on weaponized AI mentioned above.

 A few other issues are related to the assumption that economic might will convert into military advantage without discussing the scenario that military advantage translates into economic strength.

 Lastly, the analysis seems to be largely optimistic about the potential of AI while containing relatively limited considerations of the risk of AI (e.g., cognitive bias, automation bias, the limits of datafication, technological limitations) and the increasing awareness of the risk of AI among power contenders around the world. It would be helpful to situate these AI political implication discussions in more balanced discourse that considers both the potentials and concerns of AI.

 I hope these comments will be of help to the author, to whom I wish the best of luck with the next stages of work on this interesting piece!

Is the work clearly and accurately presented and does it cite the current literature?

Partly

If applicable, is the statistical analysis and its interpretation appropriate?

Yes

Are all the source data underlying the results available to ensure full reproducibility?

Yes

Is the study design appropriate and is the work technically sound?

Yes

Are the conclusions drawn adequately supported by the results?

Yes

Are sufficient details of methods and analysis provided to allow replication by others?

Partly

Reviewer Expertise:

Artificial Intelligence, Cyber Security, China Studies, Norm Diffusion, International Organisations.

I confirm that I have read this submission and believe that I have an appropriate level of expertise to confirm that it is of an acceptable scientific standard, however I have significant reservations, as outlined above.

## References

[1] : Prospects for the global governance of autonomous weapons: comparing Chinese, Russian, and US practices. *Ethics and Information Technology* .2023;25(1) : 10.1007/s10676-023-09678-x 10.1007/s10676-023-09678-x 36789353

[2] : Weaponised Artificial Intelligence and Chinese Practices of Human–Machine Interaction. *The Chinese Journal of International Politics* .2023;16(1) : 10.1093/cjip/poac024 106-128 10.1093/cjip/poac024

[3] : China’s Artificial Intelligence Ethics: Policy Development in an Emergent Community of Practice. *Journal of Contemporary China* .2022; 10.1080/10670564.2022.2153016 1-17 10.1080/10670564.2022.2153016

[4] : Great power identity in Russia’s position on autonomous weapons systems. *Contemporary Security Policy* .2022;43(3) : 10.1080/13523260.2022.2075665 407-435 10.1080/13523260.2022.2075665

[5] : Lethal Artificial Intelligence and Change: The Future of International Peace and Security. *International Studies Review* .2018;20(2) : 10.1093/isr/viy029 334-341 10.1093/isr/viy029

# Response

**Published:** 2023-07-11

**Authors:** Anupama Vijayakumar

**Affiliations:** Manipal Academy of Higher Education, India

Dear Ma'am,

 Hope you are doing well. I would like to convey my heartfelt gratitude to you for your detailed and invaluable suggestions. I am also thankful to you for sharing with me some excellent references for the purposes of revising my article. However, I do not have access to the following source through my institution or otherwise.

 Qiao-Franco G, Zhu R: China’s Artificial Intelligence Ethics: Policy Development in an Emergent Community of Practice. *Journal of Contemporary China*. 2022. 1-17

 I really think it could help me broaden my understanding on China's AI strategy (otherwise found lacking in Western sources) if I could refer to your work for the purposes of incorporating the minor revisions. I would appreciate it greatly if you could share the same with me. 

 Thanking you.

 Yours sincerely,

 Anupama

# Reviewer report: Reviewer response for version 1

**DOI:** 10.5256/f1000research.137148.r164732

**Published:** 2023-05-02

**Authors:** W Lawrence S Prabhakar

**Affiliations:** 1 Centre for Public Policy Research, Elamkulam, Kerala, India

The article "Potential impact of artificial intelligence on the emerging world order" by Anupama Vijayakumar is a comprehensive and meticulous study and analysis of the role and impact of Artificial Intelligence on the emerging world order that is driven by Technology, specifically Artificial Intelligence and its constituent forces provides a competition paradigm among the Great Powers notably USA, Russia and China.

 Artificial Intelligence itself is an Industrial Revolution that has brought about a Paradigm Shift to the nature of industrialization and specifically Defense Industrialization. The quest for primacy and dominance is cast in the most tenuous competition that sees elimination of rivals and with the augmentation of all dimensions and vistas of national power and comprehensive national power.

 The research article has well documented the cumulative impacts of the First, Second and Third Industrial Revolutions and works out the trajectories of the competing AI technologies paradigms that would eventually shape the Power primacy of each power. The author has well used the Adoption Capacity Theory to argue that the AI race is determined by strengths of relative capacity of the primary institutions, processes and their strengths and the relative disruptions it is likely to cause in the overall institutional and industrial capacities of the countries.

 The article is well annotated with facts and is salient in analysis. The critical reviews of the strengths of the AI domain capacities of the USA, Russia and China are well analysed. The references are varied and are salient by the theme and reflect the correct citations. The text of analysis is sound and is reflective of the theme. The theoretical premises are well applied and analysed and the conclusions are well drawn and meticulously placed in the correct context.

 The article could be indexed with no alterations and is most pertinent in its topic, theme and salience.

Is the work clearly and accurately presented and does it cite the current literature?

Yes

If applicable, is the statistical analysis and its interpretation appropriate?

Not applicable

Are all the source data underlying the results available to ensure full reproducibility?

Yes

Is the study design appropriate and is the work technically sound?

Yes

Are the conclusions drawn adequately supported by the results?

Yes

Are sufficient details of methods and analysis provided to allow replication by others?

Yes

Reviewer Expertise:

International Relations Theories, Technology, Foreign Policy Analysis, Strategic Studies

I confirm that I have read this submission and believe that I have an appropriate level of expertise to confirm that it is of an acceptable scientific standard.
